# Demarcating the boundary conditions of memory reconsolidation: An unsuccessful replication

**DOI:** 10.1038/s41598-022-06119-5

**Published:** 2022-02-10

**Authors:** Lotte E. Stemerding, Danielle Stibbe, Vanessa A. van Ast, Merel Kindt

**Affiliations:** Department of Clinical Psychology, University of Amsterdam, Amsterdam, The Netherlands

**Keywords:** Learning and memory, Fear conditioning, Long-term memory

## Abstract

Disrupting memory reconsolidation provides an opportunity to abruptly reduce the behavioural expression of fear memories with long-lasting effects. The success of a reconsolidation intervention is, however, not guaranteed as it strongly depends on the destabilization of the memory. Identifying the necessary conditions to trigger destabilization remains one of the critical challenges in the field. We aimed to replicate a study from our lab, showing that the occurrence of a prediction error (PE) during reactivation is necessary but not sufficient for destabilization. We tested the effectiveness of a reactivation procedure consisting of a single PE, compared to two control groups receiving no or multiple PEs. All participants received propranolol immediately after reactivation and were tested for fear retention 24 h later. In contrast to the original results, we found no evidence for a reconsolidation effect in the single PE group, but a straightforward interpretation of these results is complicated by the lack of differential fear retention in the control groups. Our results corroborate other failed reconsolidation studies and exemplify the complexity of experimentally investigating this process in humans. Thorough investigation of the interaction between learning and memory reactivation is essential to understand the inconsistencies in the literature and to improve reconsolidation interventions.

## Introduction

Maladaptive emotional memories are fundamental to the development of anxiety disorders such as specific phobias and post-traumatic stress disorder^1,2^. These memories drive persistent and unwanted behaviour that is difficult to change, such as automatic avoidance responses to harmless environmental stimuli. However, recent developments in behavioural neuroscience have revealed new ways of reducing fearful behaviour. A brief reactivation of the memory can trigger a biological process that destabilizes the memory trace, making it susceptible to change^3,4^; a process also coined as memory reconsolidation. If the memory reactivation is followed up by a pharmacological or behavioural intervention that blocks the reconsolidation of the memory, the behavioural expression of the memory is strongly reduced 24 h later. This ground-breaking discovery has been successfully translated to humans and sparked ample research into its underlying mechanisms, boundary conditions, and clinical applications^5–14^. Notwithstanding the progress that has been made in understanding the processes that drive memory reconsolidation, over the past years some fundamental observations of human fear memory reconsolidation could not always be replicated in our own lab^15,16^ and in other labs^17,18^. It appears that the success of a reconsolidation intervention is heavily dependent on the parameters of the reactivation session in interaction with learning history^9–11,19–21^. Sevenster et al.^11^ showed that the window to successfully trigger and target the reconsolidation process is narrow and easy to miss: the reactivation procedure must contain some new information to trigger the switch from stability to plasticity, but too much new information appears to prevent or undo this switch, leaving the memory in its original state. These observations are corroborated by animal studies^20,21^ and may explain why some experimental manipulations fail to induce memory reconsolidation. To understand the factors that determine the success or failure of a reconsolidation intervention, we need to get a better grasp on what are believed to be the critical conditions under which memories become susceptible to change. Consistently replicating previous results that have shed light on these conditions is an important first step in this process.

As discussed above, research in animals as well as in humans demonstrated that one critical condition for the destabilization of memories is the occurrence of an unexpected event, or prediction error (PE)^9,19^. PEs are formalized in theories of associative learning as the difference between what is occurring and what was expected^22^. Importantly, the occurrence of a PE depends not only on the characteristics of the reactivation session, but also on prior learning, as predictions are based on what was previously learned. If learning consisted of three CS presentations that were always reinforced, a reactivation session 24 h later comprising one reinforced conditioned stimulus (CS) followed by an amnesic drug (i.e., propranolol) did not affect memory expression, as no PE occurred^10^. Conversely, if only one out of three CS presentations was reinforced during learning, then one reinforced CS did trigger memory reconsolidation. It bears mentioning that the process of memory reconsolidation cannot be directly observed in humans. Reconsolidation is typically inferred from a subsequent change in memory expression (e.g., 24 h later) resulting from a previously administered amnesic drug in conjunction with memory reactivation. These findings convincingly demonstrated that the effectiveness of a reconsolidation intervention, measured by a change in fear expression one day later, depends on the occurrence of a PE. The notion of PE provides an elegant explanation for why the process of memory reconsolidation is dependent on the interaction between past learning and memory reactivation.

Even though the occurrence of a PE is an alleged necessary condition for reconsolidation to occur, it is not a sufficient condition. If multiple PEs are experienced during the reactivation session, mechanisms supporting new learning (e.g., extinction learning) may be activated, which are believed to inhibit the destabilization of the original memory^11,21^. Hence, there is both a lower and upper limit to the number of PEs necessary for reconsolidation, with the exact amount being dependent on prior learning. To gain evidence for this phenomenon, Sevenster et al.^11^ employed a fixed 50% reinforcement scheme during acquisition, in which every second trial was reinforced. This acquisition scheme across groups allowed for systematic variation in the number of PEs experienced during memory reactivation. The presentation of one unreinforced CS during reactivation caused passive retrieval (i.e., participants showed a conditioned response), but no memory reconsolidation because there were no effects of propranolol administration on fear retention at test. This can be explained by the fact that due to the 50% reinforcement rule, participants did not expect to receive an US on the first CS+ trial, and therefore a single unreinforced CS+ presentation does not constitute a PE. In contrast, two unreinforced CS presentations during reactivation (causing a single PE because the second trial was expected to be reinforced) followed by propranolol produced a strong reduction in the conditioned fear response the next day. Lastly, participants who received four unreinforced CS presentations during reactivation (i.e., multiple PEs) did not show a reduction of fear. Notably, the four unreinforced CS presentations did not result in extinction of the fear response either, indicating the existence of a state during which neither reconsolidation nor extinction processes are engaged. This intermediate state has been termed the limbo state, and it has been shown that biological processes related to reconsolidation or to extinction learning are not activated in this state^21^. These results have strongly improved our understanding of the boundary conditions of reconsolidation. Systematically manipulating the parameters of this paradigm can help to further define the exact window of opportunity for successful memory reconsolidation, and thereby may also shed light on earlier failures to replicate fundamental reconsolidation observations.

In the current study, we aimed to replicate the previous results from Sevenster et al.^11^ where a single PE following a 50% reinforcement schedule successfully triggered memory reconsolidation, while multiple PEs or the absence of a PE left the fear memory unaffected. We employed a Pavlovian fear-conditioning paradigm with one threat stimulus (CS +) and one safe stimulus (CS−). Fear responses were measured using fear-potentiated startle measurements^23^, and operationalized as differential responding between the CS+ and the CS−. During conditioning on day 1 all participants were presented with six CS+ and six CS− trials, of which the CS+ was followed by the US on every second trial. Memory reactivation took place one day later. Depending on group, the reactivation session included no PE, a single PE, or multiple PEs. All participants received a single pill of propranolol (40 mg) after memory reactivation on day 2. We then tested fear retention on day 3 and predicted that propranolol would only be effective in neutralizing the fear response in participants who received a single PE during memory reactivation. We therefore expected to observe a reduction in differential responding between the CS+ and the CS− in the single PE group at the retention test (day 3), whereas in the other two groups the differential fear response should remain intact. The current study is an exact replication of Sevenster et al.^11^, except for the conditioned stimuli, which we changed from fear-relevant spider CSs to neutral CSs. We further slightly adapted the instructions to strengthen the rule learning during acquisition. To ensure a large enough sample size, we opted for a modified Bayesian sequential testing paradigm^24^. We aimed to test a minimum of 60 participants (20 per group, which is comparable to the original sample), and for practical reasons we a priori decided to stop at a maximum of 90 participants, while performing intermittent checks every 6 participants. Importantly, the researcher collecting the data was not aware of the results of the interim analyses to avoid potential effects on experimenter behaviour^25^.

## Methods

### Preregistration

This study was preregistered at the Open Science Framework (https://osf.io/c536n/).

### Participants

The final sample consisted of ninety-one participants: no PE group (n = 30), single PE group (n = 31) and multiple PE group (n = 30). Participants were University students aged between 18 and 35 years old (mean = 20.6, SD = 2.4). Before taking part in the experiment, participants were screened to make sure they could safely take propranolol. Exclusion criteria were an anxiety sensitivity (ASI) score > 26, self-reported history of psychiatric illness, history of heart, lung, kidney or liver disorders, asthma (unless properly treated with medication), frequent (> 3 times) fainting in the past year, endocrine disorders, diabetes, epilepsy, pregnancy, the use of medication that interferes with propranolol, allergy to propranolol, and previous participation in a fear-conditioning study. Based on the US intensity rating during the calibration process, participants who rated the maximum possible intensity lower than 7 were also excluded. Further, participants were excluded if their blood pressure was lower than 100/60 or if their resting heart rate was lower than 60 beats per minute (55 when exercising > 4 h a week, 50 when intensively exercising > 7 h a week) during the blood pressure measurements on either day 1 or day 2. In line with the original study, no participants were excluded based on fear learning criteria for acquisition or extinction. Participants were randomly assigned to one of the three groups. All participants received research credits or a financial reward (€75) for their time spent in the study. The study was approved by the ethics committee of the University of Amsterdam and all experimental sessions were performed in accordance with the relevant guidelines and regulations. All participants signed informed consent before taking part.

### Conditioned stimuli

The conditioned stimuli (CSs) were two coloured (yellow and blue) fractals of size 13.5 by 13.5 cm presented on a black background on a 24-inch computer screen. The CSs were different from those used in the original experiment, which were an image of a spider and a gun (i.e., fear-relevant CSs, IAPS numbers 1200 and 6210). We ran a pilot study using the original CSs but found no evidence of differential fear acquisition. Specifically responding to the CS− was elevated compared to the NA probe, showing aberrant safety learning. Another study conducted in our lab^26^ used coloured fractals as CSs and found good fear and safety learning. As differential fear acquisition on the first day is essential to test the critical hypothesis, we have changed the CSs to fractals.

### Unconditioned stimulus

The unconditioned stimulus (US) consisted of an uncomfortable electrical stimulus (2 ms) delivered by a Digitimer DS71 (Welwyn Garden City, UK) through two 20 × 25 mm Ag/AgCl electrodes placed on the top of the wrist of the left hand. The intensity of the US was individually determined before the start of the experiment to be highly uncomfortable but not yet painful. The minimum intensity was 2 mA and the maximum intensity 70 mA.

### Fear-potentiated startle

Fear-potentiated startle (FPS) responses were measured using two 7 mm electromyography (EMG) electrodes placed on the left orbicularis oculi muscle. One ground electrode was placed on the forehead. All electrodes were filled with electrolyte gel. A loud noise (104 dB, 50 ms) was administered binaurally through headphones as startle probe. The EMG electrodes were connected to an amplifier with a 5–1000 Hz bandwidth and an input resistance of 1 GΩ. The EMG signal was recorded using the in-house VSRRP recording program. Data was sampled at 1000 Hz. The raw signal was digitized, notch filtered at 50 Hz, and bandpass filtered between 28 and 500 Hz with a 4th order Butterworth filter. Startle responses were taken as peak EMG values in a 0-200 ms window after probe onset.

### Expectancy ratings

For each trial, participants provided online expectancy ratings on an 11-point Likert scale ranging from − 5 (“I will certainly not receive an electrical stimulus”) to + 5 (“I will certainly receive an electrical stimulus”), with 0 as middle point (“I am uncertain”). Participants were instructed to rate their US expectancy by moving a cursor over the scale with the mouse, and to click to confirm. Participants had approximately six seconds to rate their US expectancy.

### Propranolol administration

Immediately after finishing the experimental task on day 2, participants orally received 40 mg of propranolol. Propranolol administration was single-blind: all participants received propranolol but were told that they could either receive propranolol or a placebo pill.

### Design and experimental task

**Figure 1:**
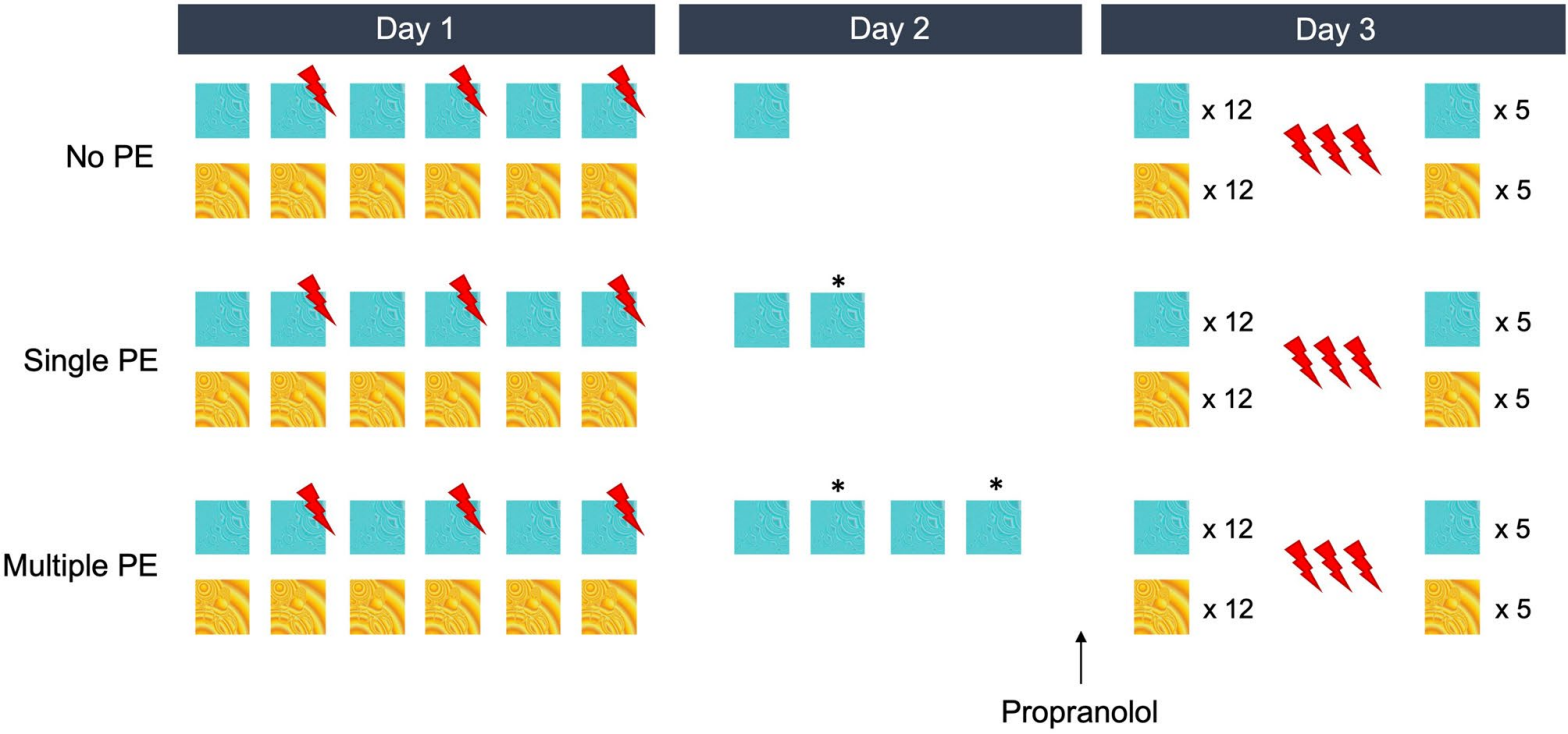
Schematic overview of the design. In this case, the blue fractal serves as the CS+ and the yellow fractal as the CS−. The * indicates the trials that are theorized to be PE trials.

We used a within- and between-subjects design, with three groups (see Fig. 1). All participants completed three sessions on three subsequent days. During the experimental task, each CS was presented on the screen for 8 s. The startle probe was presented 7 s after CS onset and the US, if presented, occured 7.5 s after CS onset. To measure baseline startle responding, startle probes were also presented alone in noise alone (NA) trials. Intertrial intervals were 15, 20, or 25 s. Each experimental session started with 10 startle probes to reduce habituation effects. The fear acquisition and extinction/reinstatement session were the same for all groups. During acquisition (day 1), the CS+, CS−, and NA probes were presented 6 times each, resulting in a total of 18 trials. The order of CS presentation was fixed for all participants (see Supplementary Fig. S1). The CS+ was reinforced on every other trial, resulting in a 50% off–on schedule. The memory reactivation phase (day 2) differed per group. The No PE group received a single unreinforced CS+ trial. Due to the 50% reinforced acquisition schedule, the first trial should be expected to be unreinforced, resulting in no PE. The single PE group received two unreinforced trials where a PE was believed to occur on the second trial, and the multiple PE group received four unreinforced trials. The number of NA trials was matched to the number of CS+ trials and no CS− trials were presented. During extinction (day 3), both CSs were presented twelve times without reinforcement, and twelve NA probes were presented. After the last extinction trial, three unexpected USs were presented to induce reinstatement of the fear response, with 15–25 s in between the last extinction trial and the first US and in between each US. After reinstatement, each CS and the NA probe was presented five more times.

### Experimental instructions

During the pilot phase it became apparent that participants had difficulty learning the contingency rule of CS+/CS− trials. Understanding the reinforcement pattern is, however, crucial for the reactivation manipulation on the second day. We have therefore adapted the experimental instructions in telling the participants that they would see two coloured images, and that one image would be followed by an electrical stimulus on *every other trial*, and the other image would never be followed by an electrical stimulus. In contrast, in the original experiment, participants were instructed that one figure would be followed by an electrical stimulus on *50% of the trials*, while the other figure would never be followed by an electrical stimulus. Instructions on day 2 and 3 were identical to the original experiment. On day 2 all participants were told to think about what they learned yesterday, and on day 3 participants were only instructed that the experiment would continue.

### Procedure

#### Day 1: fear acquisition

During the first session, subjects verbally completed a medical screening, and their blood pressure was measured. To be able to control for baseline differences in anxiety levels between the groups, participants completed anxiety sensitivity^27^ (ASI) and state and trait anxiety^28^ (STAI) questionnaires prior to the start of the experiment. State and trait anxiety may influence fear and safety learning, and anxiety sensitivity can affect the perception of the US^29,30^. Then, startle and electrical stimulus electrodes were attached. The electrical stimulus was individually calibrated to be “highly uncomfortable but not painful” by increasing the intensity from 2 milliampere (mA) with steps of 2–4 mA and asking participants to say “stop” when the electrical stimulus was highly uncomfortable. Participants rated the intensity of their choice on a scale from 0 to 10 (0 = “I did not feel anything” and 10 = “This is the most uncomfortable stimulus I can imagine to be applied via this electrode”). Participants who did not experience the maximum intensity of 70 mA as uncomfortable or difficult to tolerate (rating < 7) were excluded from the experiment. After calibration, participants were instructed both verbally by the experimenter and on screen. After finishing the fear acquisition task, participants rated the intensity of the electrical stimulus and the startle probes, and filled out a STAI-S questionnaire. All participants were instructed to sleep well, not drink or take drugs that evening, and to eat approximately 2 h before the start of the experiment the next day.

#### Day 2: memory reactivation

Approximately 24 h later, on the second day, participants returned to the lab. Participants first filled out a STAI-S questionnaire for baseline assessment, and their blood pressure and heart rate were measured. Before starting the experiment, all participants were instructed by the experimenter and again by a written text presented on the computer screen. Upon completing the memory reactivation task, all participants received a single pill of 40 mg propranolol. For safety reasons and to avoid distraction, participants remained in the lab for 90 min after pill intake. During these 90 min they were not allowed to use their phone or computer, but they could read or listen to music. At the end of the session blood pressure, heart rate, and STAI-S were measured once again.

#### Day 3: extinction and reinstatement

On the final day, again approximately 24 h later, participants returned to the lab and electrodes were placed as before. Before starting the task, participants were instructed by the experimenter and again by a written text presented on the computer screen. After finishing the extinction and reinstatement task, participants were debriefed and thanked for their time.

### Bayesian sequential updating

We made use of a modified Bayesian sequential updating paradigm^31^, with a minimum sample of 60 participants and a maximum sample of 90 participants. We performed interim analyses after reaching the minimum sample, and subsequently for every 6 participants (2 in each group). For the main effect (point 2 and 3 below) we set stopping criteria for both evidence *in favour* of an effect and for evidence *against* an effect. Data collection was terminated when all the stopping criteria below were fulfilled:

1. A Bayes factor (BF) > 10 for the difference between the mean of the last three CS+ and CS− trials during acquisition, separately in all three groups, tested by three Bayesian t-tests.
2. A BF_incl_ > 7 (in favour of an effect) or < 0.33 (against an effect) for a CS × Group interaction, comparing the CS+ vs CS− difference on the first extinction trial across all three groups in a CS (CS+, CS−) × Group (no PE, single PE, multiple PE) Bayesian repeated measures ANOVA.
3. A BF_incl_ > 7 or < 0.33 for a CS × Group interaction, comparing CS+ vs CS− difference on the first reinstatement test trial across all three groups in a CS (CS+, CS−) × Group (no PE, single PE, multiple PE) Bayesian repeated measures ANOVA.

Interim analyses were performed by a researcher (LS) who was not directly involved with participants to prevent changes in experimenter behaviour based on awareness of the direction of the effect^25^. For the results of the interim analyses, see the supplementary material S1. The final sample consisted of 91 participants because one extra participant was tested in case of an exclusion on day 1 or day 2.

### Statistical analyses

All analyses were performed in JASP^32^ (version 14.0.1). Conditioned responding was operationalized through differential FPS responses. In contrast to the preregistration, FPS values were not standardized. We decided to perform the analyses on the raw data in the current study as in the original study the data were also not standardized, and this allows for easier comparison. Nevertheless, we have also performed the analyses on the standardized data, and this did not affect the results. To test for acquisition on day 1, a Group (no PE, single PE, multiple PE) × Stimulus (CS+, CS−) × Trial (1–6) repeated measures ANOVA was performed on the FPS responses to the six acquisition trials. This differed from the interim check for the sequential updating (that did not include the factor Trial) because we did not want to risk stopping based on an overall Stimulus × Trial effect, where one group could still show poor acquisition. Further, to investigate whether the fear memories were successfully reactivated on day 2, we performed a Group (no PE, single PE, multiple PE) × Stimulus (CS+, NA) repeated measures ANOVA on the first trial of the reactivation session. Because the number of reactivation trials differ per group, we also performed two rmANOVAs on the data from the single PE and multiple PE groups only. Lastly, to investigate the expected effect of propranolol on differential FPS in the single PE group, we compared differential CS+/CS− responding on the first extinction trial (day 3) in a Group (no PE, single PE, multiple PE) × Stimulus (CS+, CS−) repeated measures ANOVA. Likewise, to test whether reinstatement was reduced in the single PE group, we ran a Group (no PE, single PE, multiple PE) × Stimulus (CS+, CS−) repeated measures ANOVA on the first reinstatement test trial (day 3).

### Bayesian statistics

All our analyses are performed using Bayesian statistics because this allowed us to use sequential testing, as well as to quantify evidence for the null hypothesis. For all ANOVAs we report inclusion Bayes factors (BF_incl_), calculated across matched models. To clarify, the BF_incl_ represents the strength of evidence in the data for the inclusion of the respective term in the model, compared against all models that do not include that term. For t-tests we report the BF_10_, which quantifies evidence against the null hypothesis. While we prefer to let the Bayes factors speak for themselves, and to not put subjective labels on them, unfamiliar readers might be helped by knowing that generally BFs > 3 are considered moderate evidence, and BFs > 10 are considered strong evidence^33^. All BFs < 1 can be interpreted as evidence in favour of the null hypothesis, where 1/BF represents the strength of evidence in favour of the null. To aid comparison with the original study we have also added frequentist statistics for each analysis.

## Results

### Baseline participant characteristics

Baseline characteristics of each group are shown in Table 1. There were no important differences at baseline between the groups, except for the gender ratio. Because sex differences may affect fear learning and retention^34^ we have performed the same analyses as below on female participants only, which did not change the pattern of results (see Supplementary Results S1).

**Table 1:** Baseline characteristics of all participants combined and within each group separately.

	All	No PE	Single PE	Multiple PE	BF_10_	*p*
N	91	30	31	30	–	–
Female/male	62/29	20/10	17/14	25/5	1.28	0.057
Age (years)	20.6 (2.4)	20.7 (2.7)	21.0 (2.5)	20.1 (1.7)	0.23	0.351
US intensity (mA)	27.6 (20.7)	30.6 (22.5)	24.2 (18.6)	28.1 (20.9)	0.18	0.480
US rating day 1	7.3 (1.4)	7.2 (1.3)	7.1 (1.6)	7.5 (1.1)	0.16	0.537
STAI-T	38.5 (8.2)	36.8 (8.2)	39.4 (8.1)	39.3 (8.4)	0.21	0.400
STAI-S	31.3 (7.1)	30.0 (6.4)	32.6 (6.8)	31.3 (8.1)	0.23	0.355
ASI	11.5 (5.6)	10.1 (5.1)	11.7 (5.4)	12.6 (6.2)	0.31	0.237

Baseline characteristics were compared between groups with a Bayesian chi-square test (sex) and a Bayesian one-way ANOVA (all other). Bayes Factors showing evidence for the existence of a difference between groups are displayed.

Successful fear acquisition in all groups.

### Successful fear acquisition in all groups

**Figure 2:**
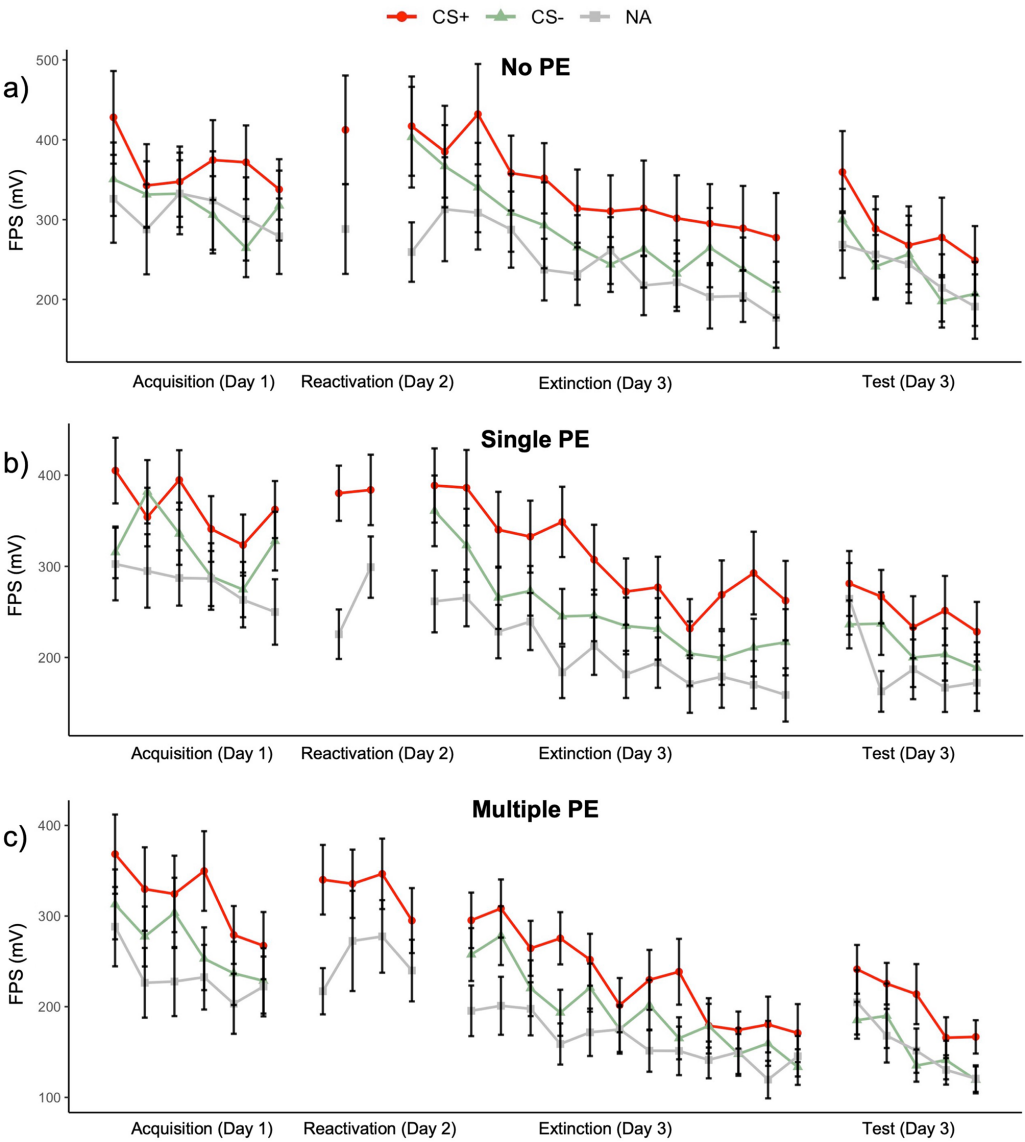
Mean fear-potentiated startle response to the CS +, CS−, and noise alone per trial in (**a**) the no PE group (n = 30), (**b**) the single PE group (n = 31), and (**c**) the multiple PE groups (n = 30). There were no significant differences in acquisition and reactivation between the groups. Against our expectations, the groups did not significantly differ on the first extinction and the first reinstatement test trial either. Error bars represent one standard error of the mean.

To investigate whether fear responses were acquired on the first day, we performed a Bayesian Stimulus (CS+, CS−) × Trial (1–6) × Group (no PE, single PE, multiple PE) repeated measures ANOVA on the FPS data (see Fig. 2). We found very strong evidence for a main effect of Stimulus (BF_incl_ = 2.80e7, F(88,2) = 30.77, *p* < 0.001, η_p_^2^ = 0.26) and of Trial (BF_incl_ = 1.45e4, F(440,5) = 7.28, *p* < 0.001, η_p_^2^ = 0.08). We find no evidence for a Stimulus × Trial interaction with Bayesian statistics but the interaction is significant with a very small effect size (BF_incl_ = 0.12, F(440,5) = 2.42, *p* = 0.035, η_p_^2^ = 0.03), indicating that overall, responding to the CS+ was larger than to the CS−, and the difference between the two stimuli slightly changed over trials. Further, we found evidence against the inclusion of a Group × Stimulus or Group × Stimulus × Trial interaction in the model (both BF_incl_ < 0.02, *p* > 0.452), showing that differential responding during fear learning did not differ between groups. The order of CS presentation during acquisition was fixed, and all participants saw the CS− first, and the CS+ second. This may explain why differential responding already occurs on the first acquisition trial, and why we find only a small effect for the expected Stimulus × Trial interaction. Moreover, in the no PE and single PE groups, CS− responding increases on the last trial, which may also affect the interaction. In general, conditioned fear responses appear to have successfully developed in all groups.

### Successful memory retrieval in all groups

To investigate whether the acquisition fear memory was successfully retrieved on day 2, we compared responding to the CS+ and the noise alone (NA) stimulus in a Stimulus (CS+, NA) × Group Bayesian rm ANOVA on the FPS data. We found strong evidence for the inclusion of the Stimulus term in the model (BF_incl_ = 3.02e9, F(88,1) = 65.34, *p* < 0.001, η_p_^2^ = 0.43) showing that responding to the CS+ was larger than to the NA (see Fig. 2). Further, we found evidence against including the Stimulus × Group term in the model (BF_incl_ = 0.12, F(88,2) = 0.32, *p* = 0.728 , η_p_^2^ < 0.01) indicating that the strength of memory retrieval did not differ across groups. Because the number of reactivation trials differed between the groups, we further tested whether the fear response to the CS+ remained elevated on the second reactivation trial in the single PE and multiple PE groups in a Type (CS+, NA) × Trial (1,2) × Group (single, multiple) Bayesian rm ANOVA. We again found very strong evidence for an effect of Stimulus (BF_incl_ = 3.65e6, F(59,1) = 42.56, *p* < 0.001, η_p_^2^ = 0.42) showing that overall responding to the CS+ was larger than to the NA. There was ambiguous evidence against the inclusion of the Stimulus × Trial term in the model, but a significant frequentist interaction (BF_incl_ = 1.21, F(59,1) = 4.60, *p* = 0.036, η_p_^2^ = 0.07), meaning that the data is slightly inconclusive from a Bayesian perspective, but it appears there is a small interaction effect. Lastly, in the multiple PE group we performed a Stimulus (CS + , NA) × Trial (1–4) Bayesian rm ANOVA to investigate whether FPS responding to the CS + would already extinguish during the reactivation session. We found evidence for an effect of Stimulus in our data (BF_incl_ = 1.37e4, F(29,1) = 14.10, *p* < 0.001, η_p_^2^ = 0.33) and evidence against the inclusion of the Stimulus × Trial term (BF_incl_ = 0.16, F(87,3) = 1.33, *p* = 0.271, η_p_^2^ = 0.04), showing that across the four reactivation trials, FPS responding to the CS+ remained consistently larger than to the NA. In sum, these results show that in all groups, the fear memory was successfully retrieved (i.e., conditioned responding to the CS+ occurs). Further, in the multiple PE group the fear response to the CS+ did not decline, showing that extinction learning did not yet take place in that group.

### No evidence for an effect of propranolol on disrupting memory reconsolidation

In line with Sevenster et al.^11^, we expected to find a lack of differential FPS responding between the CS+ and CS− on the first extinction trial on day 3, but only in the single PE group. In stark contrast with this prediction and the previous findings^11^, we found evidence against differential responding between the CS+ and CS− in all three groups (BF_incl_ for Stimulus = 0.39, F(88,1) = 1.83, *p* = 0.180, η_p_^2^ = 0.02, BF_incl_ for Stimulus × Group = 0.10, F(88,2) = 0.12, *p* = 0.883, η_p_^2^ = 0.003). Furthermore, we observe a very small main effect of Group (BF_incl_ = 1.82, F(88,2) = 3.39, *p* = 0.038, η_p_^2^ = 0.07). Visual inspection of the data (Fig. 2) shows that this lack of differential responding is driven by an increase in responding to the CS− on the first extinction trial. We cannot definitively conclude that the reconsolidation intervention was ineffective due to the lack of differential responding in the no PE and multiple PE control groups. However, because responding to the CS+ did not decrease in the single PE group, it appears unlikely that memory reconsolidation was disrupted in that group.

We further expected, based on the original study, that the return of the fear response triggered by reinstatement would be lower in the single PE group compared to the other two groups. We tested this hypothesis in a Stimulus (CS+, CS−) × Group Bayesian rm ANOVA on the first reinstatement test trial. We found strong evidence for the inclusion of the Stimulus term in the model (BF_incl_ = 38.06, F(88,2) = 12.17, *p* < 0.001, η_p_^2^ = 0.12), but in contrast with our expectations, we found evidence against the inclusion of the Stimulus × Group term (BF_incl_ = 0.09, F(88,2) = 0.10, *p* = 0.909, η_p_^2^ = 0.002). We also observe a main effect of Group (BF_incl_ = 4.18, F(88,2) = 4.42, *p* = 0.015, η_p_^2^ = 0.09). These results show that in all groups, differential responding to the CS+ returned following the unpredictable USs. Because on the graphs it appears that responding to the CS+ increased only in the no PE and multiple PE groups, and not in the single PE group, we additionally performed a repeated measures ANOVA on the last CS+ extinction trial versus the first CS+ reinstatement test trial. We found evidence for an overall effect of Trial (BF_incl_ = 7.85, F(88,1) = 8.92, *p* = 0.004, η_p_^2^ = 0.09), but not for a Trial × Group interaction (BF_incl_ = 0.22, F(88,2) = 1.05, *p* = 0.356, η_p_^2^ = 0.02), suggesting that fear returned in all three groups.

### No evidence for complete fear extinction in any of the groups

Even in the groups in which propranolol was not hypothesized to reduce the fear response at the start of day 3, extinction of the fear response should occur towards the end of the extinction session. However, we found no evidence of a reduction in the differential fear response throughout the session (see Supplementary Results S1), which suggests that normal extinction learning mechanisms were ineffective in all groups.

### No evidence for a decline in expectancy ratings on day 3

**Figure 3:**
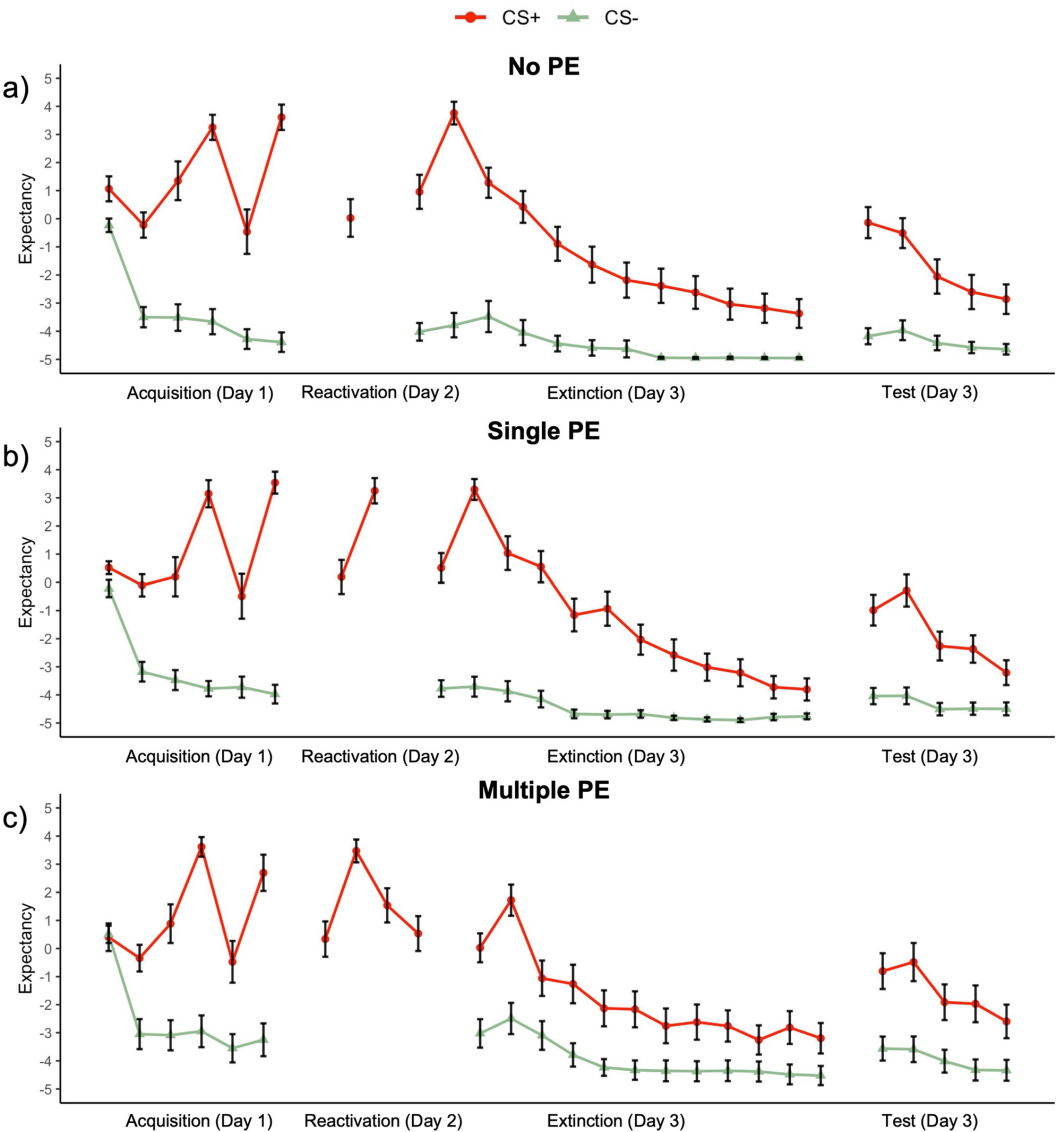
Mean US expectancy ratings to the CS+, CS−, and noise alone per trial in (**a**) the no PE group (n = 30), (**b**) the single PE group (n = 31), and (**c**) the multiple PE group (n = 30). Error bars represent one standard error of the mean.

The occurrence of a PE is assumed to be a necessary condition for memory reconsolidation. One crucial finding from Sevenster et al.^11^ was that expectancy ratings declined from the last acquisition trial to the second extinction trial in the single and multiple PE groups, indicating that new information regarding the CS–US contingencies has been learned during memory reactivation. In the no PE group the ratings did not change. This shows that a change in expectancy ratings may be used as an indirect index of PE. Due to the 50% reinforcement rule, we expected to find a decrease in expectancy ratings only on the second trial of extinction. With three Bayesian paired t-tests (one for each group) we tested whether US expectancy (Fig. 3) on the second extinction trial differed from the last acquisition trial. As expected, in the no PE group, we found moderate evidence against a difference in US expectancies (BF_01_ = 5.02, t(29) = 1.19, *p* = 0.245, d = 0.22). However, in both the single PE group (BF_01_ = 4.64, t(30) = 0.74, *p* = 0.467, d = 0.13) and the multiple PE group (BF_01_ = 2.64, t(29) = 1.50, *p* = 0.114, d = 0.27) we also found evidence against a difference between US expectancies on the last acquisition trial versus the second extinction trial. These results show that in none of the groups the US expectancy ratings declined due to the non-reinforced reactivation trials, which may indicate that our PE manipulation was ineffective and that the memories were not successfully destabilized.

### Exploratory analysis: include only participants who updated CS–US contingency on day 3

Given that the previous analysis demonstrates that a change in expectancy ratings did not occur in any of the groups, it appears that participants may not have experienced a PE, even in the intended single PE group. Therefore, we exploratorily compared differential FPS responding at the start of extinction between participants in the single PE group who did show a decrease in US expectancy to participants who did not. We calculated an expectancy change score by subtracting the US expectancy rating on the second extinction trial from the rating on the last acquisition trial and marked participant as “Change” when this difference score was larger than 0 (i.e., expectancy was lower on the second extinction trial; 13 participants), and as “No Change” when the score was smaller or equal to 0 (18 participants). Similar to the main analyses, we found weak evidence against a CS+/CS− difference on the first extinction trial (BF_incl_ for stimulus = 0.35, F(29,1) = 0.83, *p* = 0.371, η_p_^2^ = 0.03) for both the Change and the No Change group (BF_incl_ for a Stimulus × Group interaction = 0.38, F(29,1) = 0.31, *p* = 0.584, η_p_^2^ = 0.01). It bears mentioning that it is currently unknown (and likely dependent on the strength of prior learning) what amount of expectancy updating may reflect the occurrence of a necessary and sufficient PE in order to trigger the process of memory reconsolidation. The division between Change and No Change participants in this exploratory analysis is thus rather arbitrary, but it at least shows that the pattern in the data does not seem to differentiate between people who either decreased their outcome expectancy or who increased their expectancies or did not change at all.

### Further exploratory analyses: the effect of US intensity and sex

We further explored potential effects of two main differences between the current and the original sample (see Supplementary Table S1). On average, the US intensity and the number of men were significantly larger in the current sample. To exclude the possibility that these variables affected the results, we ran the main analyses again but now including US intensity and sex as a covariate and a between-subjects factor respectively (see Supplementary Results S1). There were no effects of these variables, and we therefore conclude that these differences in sample characteristics are not the (main) reason why we did not replicate the original effects.

### The effect of propranolol on blood pressure, heart rate and STAI-S scores

Propranolol is a beta blocker that is known to lower heart rate and blood pressure. To ensure that propranolol has been effective in inducing these changes, we compared blood pressure, heart rate and state anxiety scores before and 90 min after propranolol intake, and we indeed found the expected decline in all three measures (see Table 2). Due to the lack of a placebo group, we cannot firmly conclude whether these pre-to-post differences are caused by propranolol or by the 90 min of rest. However, the size of the pre to post differences in blood pressure and STAI-S scores were comparable to the original study (see Supplementary Table S2), and we therefore believe that propranolol has been effective in inducing these well-known physiological effects.

**Table 2:** Blood pressure, heart rate and state anxiety at the start of the day 2 session versus 90 min after propranolol intake.

Measure	Start of D2	End of D2	BF_10_	*p*
STAI-S	31.6 (8.6)	28.6 (6.6)	7.4e19	< 0.001
Blood pressure (Sys)	115 (10.0)	104 (10.1)	1.5e10	< 0.001
Blood pressure (Dias)	72 (6.1)	67 (6.7)	1.3e32	< 0.001
Heart rate	82 (14.4)	57 (9.6)	3604	< 0.001

## Discussion

In the current study we aimed to replicate the results from Sevenster et al.^11^ who showed that administration of propranolol after a reactivation session including a single PE disrupted memory reconsolidation and reduced fear memory retention one day later. We did not observe differential responding between the CS+ and the CS− on the first extinction trial regardless of group, showing no evidence of memory retention in either of the groups. This prohibits us from testing our initial prediction that differential responding would only decrease in the single PE group. However, we believe the lack of differential responding on the first extinction trial is largely attributable to elevated responding to the CS−, perhaps due to uncertainty or generalization, rather than decreased responding to the CS+. Therefore, we interpret the lack of differences between groups as evidence against an effect of propranolol in any of the groups, demonstrating that the manipulation designed to disrupt fear memory reconsolidation was ineffective. Importantly, similar to previous unsuccessful reconsolidation studies^15,16^, some basic aspects of associative fear learning such as differential fear responding at the start of the retention test (i.e., day 3) and a reduction of fear during extinction were not very strong in this study. Therefore, the conclusions that we can draw from the current results are limited.

Disrupting memory reconsolidation with propranolol is believed to be a promising new method to reduce automatic fear responses in humans^5,6,8,10,11,35^. However, in line with the results presented here, many others have not been able to replicate effects of interfering with memory reconsolidation^15–18,36^. Importantly, disrupting memory reconsolidation comprises two independent events: first, the memory should be successfully destabilized, meaning that following a reactivation procedure, the memory undergoes the biological transition from stability to plasticity. Second, propranolol (or any other amnesic agent) must interfere with the restabilization process. Both processes are necessary for a successful manipulation of the fear response, which implies that either one or both processes failed to be engaged in the current study. Unfortunately, it is not possible to independently test the occurrence of destabilization and restabilization in humans, as there currently exist no behavioural read-outs of either of these processes independent from the obvious success of the entire manipulation that is typically observed 24 h later. We can therefore only speculate why our manipulation was ineffective in neutralizing the fear memory.

Based on the FPS data we believe that the memory was successfully retrieved during the reactivation session (i.e., day 2), because conditioned responding to the CS+ was significantly larger than to the NA probe. However, the observation of a fear response during reactivation does not guarantee the destabilization of the memory^9,21,37,38^. For destabilization to take place, memory reactivation needs to trigger the experience of a PE or another unexpected event^9,10,39^. While there are no direct behavioural measures of PE occurrence, it has been suggested that a change in expectancy ratings can be used as an indirect index in reconsolidation studies^10,11^. In other words, a significant decrease in US expectancy from the end of acquisition to the start of extinction, driven by the reactivation session, was previously observed in the single PE group where reconsolidation occurred successfully. Critically, we did not observe these changes at the group level in the single PE group, suggesting that the memory reactivation session may have failed to induce a PE and subsequent memory destabilization. However, when we selected only those participants who lowered their US expectancy after the critical manipulation, we did not observe the expected effects of blocking reconsolidation either. This suggests that our unsuccessful replication is not only caused by a failure to induce a decrease in the participants’ outcome expectancy—deemed to reflect PE—but also a general failure to trigger destabilization. In sum, based on the absence of the expected reduction in US expectancy during the critical PE manipulation we believe that the manipulation in the current study was less effective than in the original study. However, our exploratory analyses suggest that we cannot ascribe our null results entirely to a failed PE manipulation. Other unsuccessful examples of memory reconsolidation also included PE in the reactivation session^15–17^, which demonstrates that PE occurrence is not sufficient for destabilization. There thus appear to exist other factors that can prevent memory destabilization even in the presence of a PE. To improve our understanding of the exact role of PE in memory destabilization, we need robust behavioural indices that may either better quantify PE occurrence or otherwise indicate memory destabilization. The current lack of such indices prohibits us from strongly concluding whether the results of this study are due to an unsuccessful PE manipulation or to a more general failure to destabilize fear memory.

With regards to the effectiveness of propranolol in preventing the restabilization of the fear memory, we show that in all groups, blood pressure and heart rate decrease 90 min after propranolol intake, which is in line with the standard physiological effects of propranolol. The lack of a placebo control group makes conclusive interpretation of these changes in blood pressure and heart rate difficult, but the observed physiological effects are comparable to the previous study in which propranolol did disrupt reconsolidation^11^. Based on the physiological effects, we assume that propranolol was active during the potential reconsolidation window and would have been successful in disrupting restabilization if the memory had in fact been destabilized. We therefore believe that the lack of effects that are expected from a reconsolidation intervention are due to unsuccessful destabilization of the target memory rather than to unsuccessful drug effects.

While known boundary conditions of reconsolidation can explain some null findings in the literature, we have directly replicated a highly successful study from our own lab, and thus used parameters that we know worked well in the past. The current study was, however, different from the original study in two aspects: we replaced the fear-relevant CSs (spider pictures) with neutral CSs (coloured fractals), and we changed the instructions on the first day to strengthen the rule learning. Replacing the fear-relevant stimuli with neutral stimuli served to increase the eligible sample, as it is not necessary to exclude participants with a pre-existing high fear of spiders when using neutral CSs. Furthermore, results from several pilot studies showed better fear acquisition using neutral CSs. Because the interpretation of our results is entirely dependent on the acquisition of a fear response on the first day, we used neutral stimuli in the current replication study. One may argue that this change could have affected the results, because it is the main difference between the original and the current experiment. However, although we cannot with certainty say that our results would have been the same when using fear-relevant stimuli, previous studies have compared fear-relevant versus fear-irrelevant stimuli in a reconsolidation paradigm and found no differences between the groups^18,40^. Moreover, reconsolidation studies using fear-irrelevant stimuli have also been successful in the past^41^, suggesting that reconsolidation is not dependent on the fear-relevance of the conditioned stimuli.

The second procedural change also followed from the pilot study where we observed that participants had difficulties learning the 50% reinforcement rule when using the original instructions. Critical to the manipulation of no PE and single PE, the participants should remember the contingency schedule of the acquisition and subsequently expect that the first reactivation trial is unreinforced. For this reason, we emphasized that the reinforcement of the CS+ would occur on every other trial, whereas in the original instructions, participants were only told that the reinforcement would be 50%. Visual inspection of the graphs indeed suggests that the rule learning was more pronounced in the current study than in the original study since expectancy ratings in the current study are more in line with the actual reinforcement pattern. The stronger focus on rule learning could, however, have prohibited destabilization of the original memory as large structural (i.e., rule violating) PEs are suggested to signal the formation of a new memory^42^. Latent cause theory proposes that during associative learning, latent (hidden) causes are inferred that drive the predictive relationship between the CS and the US^43^. Knowledge about which latent cause is active can be used to generate predictions about the variables in the environment (i.e., whether the CS predicts the US). The relationship between CS and US within a latent cause is updated by experienced PEs, but these same PEs are also used to guide structural learning and determine which latent cause is active. Large PEs may signal the formation of a new latent cause. Importantly, it was proposed that the likelihood of the original latent cause being active determines whether the original memory is destabilized (and thus susceptible to manipulations) or whether a new memory is formed^42^. The occurrence of a large structural PE may signal that a new latent cause is active, and subsequently the original memory is not destabilized. We speculate that due to the strong focus on rule learning on the first day, the PE that occurred during reactivation may have caused the formation of a new memory, rather than the updating of the original CS–US relationship, which may explain why propranolol administration did not affect subsequent fear memory expression.

Another fundamental limitation of the current study is that the fear responses we observed are different to those observed in the original study^11^. For example, unlike the original study, in all three groups we observed poor extinction learning. Aberrant learning on the third day has previously been observed in failed reconsolidation studies in humans^15^ as well and may indicate that different learning mechanisms are at play. It is known that stronger fear memories are resistant to reconsolidation interference^44–46^ and a recent study showed that in rats, these strong memories also display poor extinction^46^. This suggests that the fear memories in the current study may have been too strong to be susceptible to memory reconsolidation, and that incomplete extinction could serve as a potential indicator of this boundary condition. These findings also illustrate the complex interaction between the necessity to obtain strong fear acquisition and subsequent differential fear retention on the one hand, and the subtle manipulations necessary to trigger reconsolidation on the other hand. What occurs during a reactivation procedure is highly dependent on what was learned during acquisition, and it appears that the requirement for strong learning to test the reconsolidation hypothesis may in some cases impede the engagement of the reconsolidation process under investigation.

Further, we find no differential fear response at the start of extinction in all groups. Technically, this may indicate that memory expression was in fact reduced in all groups, and this would leave us with no control group to test our predictions against. However, based on the data of the other extinction trials, we do believe that the fear memory was retained in all groups. Collapsed over all extinction trials, we found a very strong main effect of stimulus type, showing that FPS responses were larger for the CS+ compared to the CS− in all three groups. Further, FPS responding to the CS+ did not reduce from the last acquisition trial to the first extinction trial (see Supplementary Results S1). We therefore believe that the absence of a differential conditioned response on the first extinction trial is likely caused by uncertainty concerning the CS− on the first extinction trial or by high generalization across stimuli. Notably, during the reactivation session on the second day the CS− was not presented in any of the groups, which may have increased the ambiguity of the stimulus during the first presentation on the third day. While there is little known about the effect of uncertainty on startle responses^47^, one study showed that responses tend to be larger for unpredictable threat^48^. Alternatively, the use of neutral stimuli may have increased fear generalization on the third day, or participants may have been less able to distinguish between the fear-irrelevant neutral stimuli than between the spider stimuli used in the original study. In any case, the fact that we did observe differential responding collapsed over all trials of the extinction phase, and no effects of group, suggests that memory expression was intact in all three groups.

Lastly, our sample differed slightly from the original sample (see Supplementary Table S1). For one, the US intensity is significantly higher in the current sample. The same work-up procedure was used, in which the participants were exposed to increasing levels of the stimulus and had to say stop when the stimulus was “clearly uncomfortable”. Although it is unclear whether participants indeed subjectively experienced the US as more aversive than in the original study, the higher US intensities could have caused stronger fear learning. On the other hand, it also means that participants were exposed to more US presentations before starting acquisition, which may strengthen habituation and would weaken fear learning. Either way, we tested the possibility that the US intensities affected the data but including the US intensity as a covariate did not change the results. Hence our null findings seem not to be attributable to the higher US intensities administered in the current study. Another important difference is that the current sample consisted predominantly of international students compared to Dutch students in the original sample. The experiment was therefore conducted in English, which is not the native language of most participants.

In conclusion, there are a substantial number of studies in both animals and humans showing a strong reduction of fear after disrupting reconsolidation, yet other studies, including the current one, have failed to replicate these results. This discrepancy between highly successful studies, including neurobiological studies in animals, and the failed replications is puzzling. Key to the success of a reconsolidation procedure is the destabilization of the original memory trace, which occurs only under specific conditions. Violations of these conditions may explain some of the failed replications, yet here we have tested a reconsolidation manipulation using a paradigm that we know was previously effective both in animals^21^ and humans^11^. We have exploratively tested several potential explanations for our results, but without success. Our findings thus suggest that either the parameters that drive reconsolidation may be even more complex than was previously believed, or that the reductions in conditioned fear responding demonstrated by other studies were produced by processes other than reconsolidation interference. To answer these questions, and to improve our knowledge of the parameters that drive reconsolidation, we need an index of memory destabilization that is independent of the success of the entire procedure. Some progress has been made in understanding the neurobiological processes that govern destabilization, and a few studies have explored neural signatures of reconsolidation using fMRI^14,49,50^. Further investigation of the brain mechanisms supporting reconsolidation is invaluable to improve both our mechanistic understanding of reconsolidation as well as its boundary conditions^51^. However, the development of a precise and accessible behavioural or physiological marker of destabilization that can be used in behavioural studies is key to large scale clinical applications. Until then, we should continue to systematically vary the reactivation conditions, replicate prior research, and publish the outcomes of these studies regardless of the observed effects. Here we show that even an almost exact replication of a previously successful paradigm is no guarantee for observing a reconsolidation effect. To understand these inconsistent findings, it is crucial to further identify the precise conditions under which memories reconsolidate, especially given the exciting and promising opportunity that memory reconsolidation offers for the treatment of fear and anxiety disorders.

## Supplementary Information

Supplementary Information.
