# Supplementary material for: Demarcating the boundary conditions of memory reconsolidation: An unsuccessful replication
Source: Sci Rep. 2022 Feb 10;12:2285. doi: 10.1038/s41598-022-06119-5 (PMC8831535; doi:10.1038/s41598-022-06119-5)
Supplement: Supplementary file 1 — Supplementary Information. [file 41598_2022_6119_MOESM1_ESM.pdf]

Supplementary Material

An unsuccessful replication attempting to demarcate the boundary conditions of memory reconsolidation

Lotte E. Stemerding, Danielle Stibbe, Vanessa A. van Ast, Merel Kindt

Supplementary Figures and Tables

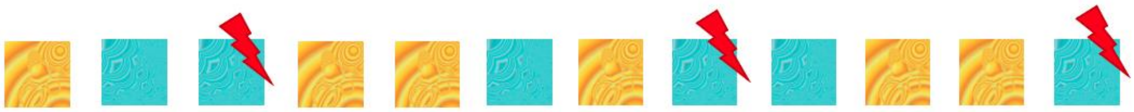

Supplementary Figure 1. The order of stimulus presentation, which was fixed for all participants. Assignment of each colored fractal to the CS+ and CS- was counterbalanced, here only one version is shown where the CS+ is the blue fractal and the CS- is the yellow fractal.

	**Current sample**	**Sevenster (2014)**	**BF_{10}**
N	91	52	-
Female/Male	62/29	41/11	0.48
Age (years)	20.6 (2.4)	21.1 (2.8)	0.38
STAI-T	38.5 (8.2)	36.7 (8.5)	0.38
ASI	11.5 (5.6)	9.9 (5.4)	0.64
US intensity	27.6 (20.7)	15.0 (8.4)	404.8

Supplementary Table 1. Sample characteristics of the current versus the original sample, and the BFs showing the evidence for a difference between the samples on each of the characteristics. All data are shown as mean (s.d.).

	**Current sample**				**Sevenster (2014)**			
	t = 0	t = 1	*p*	η^2_p	t = 0	*t = 1*	*p*	η^2_p
Systolic	115.2 (10.0)	103.9 (10.1)	< .001	.67	113.9 (13.1)	99.6 (12.2)	< .001	.61
Diastolic	71.8 (6.1)	66.6 (6.7)	< .001	.44	68.0 (8.3)	65.3 (8.7)	< .01	.13
Heart rate	82.0 (14.4)	57.4 (9.6)	< .001	.82	n/a	n/a	-	-
State anxiety	31.6 (8.6)	28.6 (6.6)	< .001	.21	33.8 (6.9)	32.6 (9.9)	< .001	.19

Supplementary Table 2. Differences in blood pressure, heart rate and state anxiety pre and post propranolol intake. To allow for a direct comparison of effect sizes, the analyses were performed using frequentist statistics. All data are shown as mean (s.d.).

Supplementary Results

Main analyses repeated including gender as between-group factor

To control for gender differences we added gender as between-group factor to our main analyses. To test for acquisition, we performed a Gender (male, female) × Group (no PE, single PE, multiple PE) × Stimulus (CS+, CS-) × Trial (1-6) Bayesian repeated measures ANOVA. Similar to the main analysis, we found a main effect of Stimulus (BF_{incl} = 2.82e7, F(85,1) = 23.02, $p < .001$, $\eta^2_p = .21$) and Trial (BF_{incl} = 1.30e4, F(425,5) = 4.73, $p < .001$, $\eta^2_p = .05$), but evidence against a Stimulus x Trial interaction (BF_{incl} = 0.12, F(425,5) = 1.96, $p = .084$, $\eta^2_p = .02$). We found no evidence for an effect of Gender (all $BF_{incl} < 0.15$, $p > .472$). To test for an effect of propranolol we performed a Stimulus (CS+, CS-) × Gender × Group Bayesian rm ANOVA on the first extinction trial. We found evidence against an effect of Stimulus (BF_{incl} = 0.37, F(85,1) = 1.26, $p = .265$, $\eta^2_p = .02$) or a Stimulus × Group interaction (BF_{incl} = 0.11, F(85,2) = 0.02, $p = .982$, $\eta^2_p < .01$). We found no effects of or interactions with Gender (all $BF_{incl} < 0.23$, $p > .747$). On the first reinstatement test trial we found evidence for an effect of Stimulus (BF_{incl} = 36.05, F(85,1) = 7.17, $p = .009$, $\eta^2_p = .08$) but against a Stimulus × Group interaction (BF_{incl} = 0.10, F(85,2) = 0.27, $p = .767$, $\eta^2_p < .01$). We found inconclusive or no effects of or interactions with Gender (all $BF_{incl} < 0.78$, $p > .153$). The results from these extra analyses are comparable to our main findings.

Main analyses repeated including US intensity as covariate

To control for possible effects of US intensity on our results, we have added this variable as a covariate to our analyses. To test for acquisition, we performed a Group (no PE, single PE, multiple PE) × Stimulus (CS+, CS-) × Trial (1-6) Bayesian repeated measures ANOVA with US intensity as covariate. Similar to the main analysis, we found a main effect of Stimulus (BF_{incl} = 2.80e7, F(87,1) = 12.71, $p < .001$, $\eta^2_p = .13$) and Trial (BF_{incl} = 1.49e4, F(435,5) = 4.32, $p < .001$, $\eta^2_p = .05$), but evidence against a Stimulus × Trial interaction (BF_{incl} = 0.12, , F(435,5) = 0.75, $p = .585$, $\eta^2_p < .01$). We found no evidence for an effect of US intensity ($BF_{incl} < 0.28$, $p > .723$) or of any effects of Group (all BF_{incl} < 0.28, $p > .458$). To test for an effect of propranolol we performed a Stimulus (CS+, CS-) × Group Bayesian rm ANOVA with US intensity as covariate on the first extinction trial. We found weak

evidence against an effect of Stimulus (BF_{incl} = 0.38, F(87,1) = 0.07, p = .788, η^2_p < .01) or a Stimulus × Group interaction (BF_{incl} = 0.10, F(87,2) = 0.15, p = .864, η^2_p < .01), and weak evidence against an effect of US intensity (BF_{incl} = 0.67, F(87,1) = 0.44, p = .511, η^2_p < .01). On the first reinstatement test trial we found evidence for an effect of Stimulus (BF_{incl} = 36.03, F(87,1) = 7.21, p = .009, η^2_p = .08), but against a Stimulus x Group interaction (BF_{incl} = 0.11, F(87,2) = 0.14, p = .872, η^2_p < .01). We also found no evidence of an effect of US intensity (BF_{incl} = 0.52, F(87,1) = 0.59, p = .445, η^2_p < .01). The results from these extra analyses are thus comparable to our main findings, and we find no effects of US intensity.

Differential responding on all extinction trials

To investigate differential responding on all extinction trials and to test for an extinction effect, we performed a Stimulus (CS+, CS-) × Trial (1-12) × Group Bayesian rm ANOVA. We found strong evidence for an effect of Stimulus (BF_{incl} = 5.49e18, F(88,1) = 30.87, p < .001, η^2_p = .26), showing that collapsed over all trials responding to the CS+ was larger than to the CS-. We further found evidence for an effect of Trial (BF_{incl} = 3.54e55, F(968,11) = 29.45, p < .001, η^2_p = .25), showing that for both the CS+ and the CS- FPS responding decreased. However, we found strong evidence against a Stimulus × Trial interaction (BF_{incl} < 0.01, F(968,11) = 0.72, p = .722, η^2_p < .01) showing that differential responding did not decrease over the trials. Lastly, there was strong evidence against an interaction effect of Group (all BF_{incl} < 0.04, p > .516). These data show that while there was no difference between CS+ and CS- responding on the first extinction trial, fear retention appears to be quite strong across all extinction trials, with all groups showing poor extinction.

Responding to the CS+ on the last acquisition versus the first extinction trial

To test whether responding to the CS+ decreased on the first extinction trial compared to the last acquisition trial, we performed a Trial (last acquisition, first extinction) × Group Bayesian rm ANOVA. We found evidence for a main effect of Trial (BF_{incl} = 1.27, F(88,1) = 4.61, p = .035, η^2_p = .05), but in absolute numbers responding on the first extinction trial was *larger* than on the last

acquisition trial (see main document Figure 2). We found inconclusive evidence for an effect of Group (BF_{incl} = 1.07, F(88,2) = 2.76, *p* = .069, η^2_p = .06).

Results from the intermittent checks in the Bayesian sequential updating paradigm

Check 1, N = 60 (20 per group), 01/03/2021

We found evidence for differential responding at the end of acquisition (last three CS+ trials versus last three CS- trials) in the no PE group (BF_{10} = 7.65) and the multiple PE group (BF_{10} = 1463.48), but not in the single PE group (BF_{10} = 1.15). The Stimulus (CS+,CS-) × Group rmANOVA showed support against an Stimulus x Group interaction on the first extinction trial (BF_{incl} = 0.23) and the first reinstatement trial (BF_{incl} = 0.26). Data collection was continued because we did not reach the stopping criteria (BF_{10} > 10) for acquisition in the no PE and single PE group.

Check 2, N = 66 (22 per group), 18/03/2021

We found evidence for differential responding at the end of acquisition (last three CS+ trials versus last three CS- trials) in the no PE group (BF_{10} = 6.20) and the multiple PE group (BF_{10} = 2735.52), but not in the single PE group (BF_{10} = 0.87). The Stimulus (CS+,CS-) × Group rmANOVA showed support against an Stimulus x Group interaction on the first extinction trial (BF_{incl} = 0.21) and the first reinstatement trial (BF_{incl} = 0.20). Data collection was continued because we did not reach the stopping criteria (BF_{10} > 10) for acquisition in the no PE and single PE group.

Check 3, N = 72 (24 per group), 01/04/2021

We found evidence for differential responding at the end of acquisition (last three CS+ trials versus last three CS- trials) in the no PE group (BF_{10} = 6.60) and the multiple PE group (BF_{10} = 123.90), but not in the single PE group (BF_{10} = 0.49). The Stimulus (CS+,CS-) × Group rmANOVA showed support against an Stimulus x Group interaction on the first extinction trial (BF_{incl} = 0.26) and the first reinstatement trial (BF_{incl} = 0.27). Data collection was continued because we did not reach the stopping criteria (BF_{10} > 10) for acquisition in the no PE and single PE group.

Check 4, N = 78 (26 per group), 13/04/2021

We found evidence for differential responding at the end of acquisition (last three CS+ trials versus last three CS- trials) in the no PE group (BF_{10} = 19.5) and the multiple PE group (BF_{10} = 86.9), but not in the single PE group (BF_{10} = 0.53). The Stimulus (CS+,CS-) × Group rmANOVA showed support against an Stimulus x Group interaction on the first extinction trial (BF_{incl} = 0.17) and the first reinstatement trial (BF_{incl} = 0.24). Data collection was continued because we did not reach the stopping criteria (BF_{10} > 10) for acquisition in the single PE group.

Check 5, N = 84 (28 per group), 23/04/2021

We found evidence for differential responding at the end of acquisition (last three CS+ trials versus last three CS- trials) in the no PE group (BF_{10} = 54.20) and the multiple PE group (BF_{10} = 155.70), but not in the single PE group (BF_{10} = 1.04). The Stimulus (CS+,CS-) × Group rmANOVA showed support against an Stimulus x Group interaction on the first extinction trial (BF_{incl} = 0.16) and the first reinstatement trial (BF_{incl} = 0.16). Data collection was continued because we did not reach the stopping criteria (BF_{10} > 10) for acquisition in the single PE group.

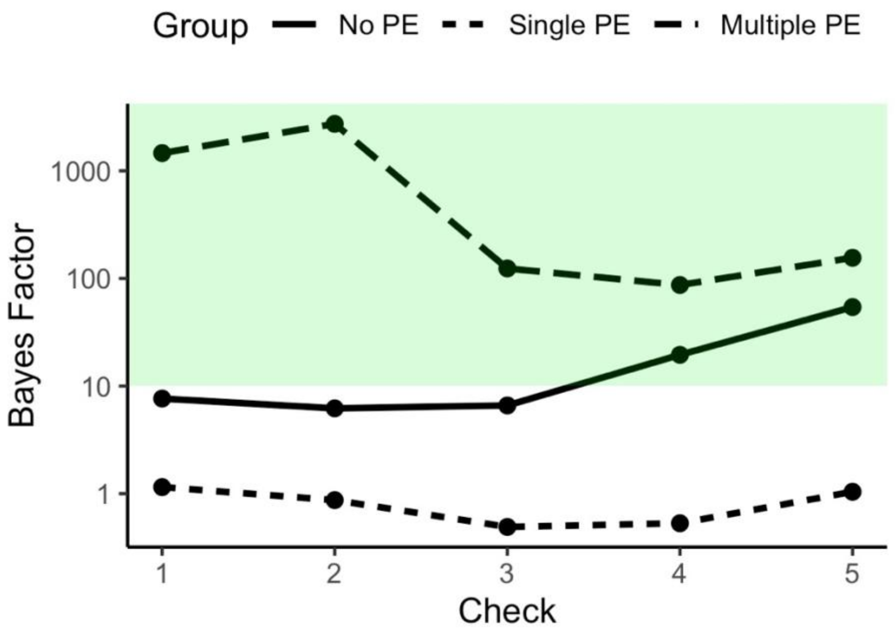

Supplementary Figure 2. Bayes factors of the acquisition test for each of the groups at every check. Green shaded area represents the stopping criterium for acquisition (BF > 10). Scale on the y-axis is logarithmic for visualization purposes.

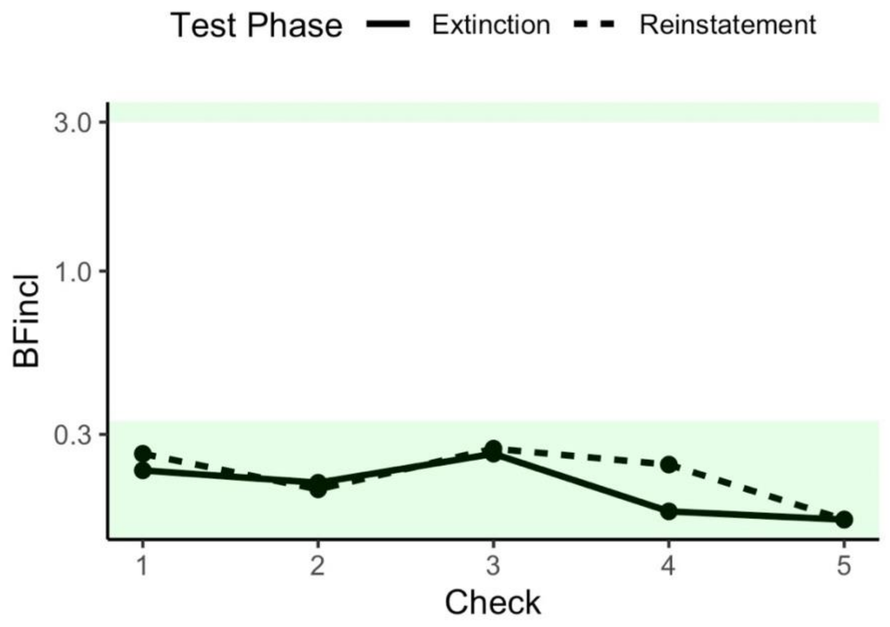

Supplementary Figure 3. Bayes factors of the extinction and reinstatement test (this test included a factor “Group”) at every check. Green shaded areas represent the stopping criteria in favor of the null (< 0.33) and against the null (> 3.0).
